# Supplementary material for: Knowledge of blood loss at delivery among postpartum patients
Source: PeerJ. 2016 Aug 31;4:e2361. doi: 10.7717/peerj.2361 (PMC5012285; doi:10.7717/peerj.2361)
Supplement: Supplemental Information 2 [file peerj-04-2361-s002.docx]

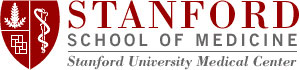


**Survey to assess obstetric patients’ knowledge of hematologic and hemorrhagic-related adverse outcomes with vaginal or cesarean delivery**

Please indicate which is the most appropriate answer to each question either by ticking the box or circling the correct word or phrase. If you have any questions about the survey, a member of the research team will be happy to provide help.

**Your Background/Pregnancy**

**1.      Please choose one option to indicate your highest level of education:**

Less than college: 🞏

College degree: 🞏

Graduate degree: 🞏

**2.      Please indicate what is your annual household income:**

Less than $10,000: 🞏

Between $10,000 and $49,000: 🞏

Equal to or greater than $50,000: 🞏

**3.      What is your marital status?**

Married / Living with partner 🞏

Unmarried, Living with other adults 🞏

Unmarried, Living without other adults 🞏

Refused to answer / unknown 🞏

**4.      Did you take prenatal iron and folate prior to delivery? Yes / No**

**Questions about bleeding during delivery**

**5.      What is the normal volume of blood loss that occurs during a normal vaginal delivery or cesarean delivery?**

(Patients will be shown pictures of common volumes e.g. can of coca-cola; bottle of wine to help gauge volume)

Vaginal delivery: ____________ (ml)

Cesarean delivery: ____________ (ml)

**6.    A) Were you informed by a member of your care team about the quantity of blood you lost during the delivery?**

Yes /No

**B) How would you describe the volume of blood lost during your most recent delivery?**

Mild 🞏

Moderate 🞏

Severe 🞏

**C) Can you provide an estimate (in mls) of the volume of blood loss during your delivery?**

_____ mls

**7.   If you have undergone prior deliveries (vaginal or cesarean), do you recall experiencing a postpartum hemorrhage with any prior deliveries (vaginal or cesarean)?**

Yes / No

If No, move to question 8.

If Yes, please indicate the total number of episodes of postpartum hemorrhage: _______

**8.   Were you aware about the risks associated with major bleeding (such as transfusion) prior to delivery?**

Yes / No

If No, move to question 8.

If Yes, please indicate the total number of episodes of postpartum hemorrhage: _______

**A) Did you read or receive information about any of the following risks:**

Risk of receiving wrong blood: YES / NO

Risk of infection from bacteria: YES / NO

Risk of Hepatitis: YES / NO

Risk of HIV: YES / NO

**B) Did a healthcare professional provide information about transfusion risk? YES / NO**

If yes, who was the healthcare professional? (Circle as appropriate)

Obstetrician / Anesthesiologist / Nurse / Other

1. **Did you acquire information about major bleeding and/or transfusion related to delivery from the following information sources:**

Health or Pregnancy related Website: YES/NO

Social Media (e.g., facebook): YES / NO

Newspaper/Radio/TV: YES/NO

Information Leaflet: YES/NO

Friends/Family/Other pregnant women: YES/NO

1. **Please answer the following questions about the quality of information that you have received about bleeding and the use of a blood transfusion? Tick the best answer.**
2. **‘I was very aware that vaginal bleeding occurs during and after delivery’**

Completely agree 🞏

Somewhat agree 🞏

Neither agree nor disagree 🞏

Somewhat Disagree 🞏

Totally disagree 🞏

1. **‘I was fully aware about the risk of major bleeding (postpartum hemorrhage) related to my delivery’**

Completely agree 🞏

Somewhat agree 🞏

Neither agree nor disagree 🞏

Somewhat disagree 🞏

Totally disagree 🞏

1. **What has been the quality of information that you have received about the use of a blood transfusion related to delivery and the period after delivery? Tick the best answer.**

|  | Tick box with the most appropriate answer below: |
| --- | --- |
| The information about blood transfusions was explained clearly and was very understandable. |  |
| The information about blood transfusions was incompletely or inadequately explained but I have a reasonable level of understanding. |  |
| The information was poorly explained and I have a limited understanding about blood transfusions. |  |
| The information was not explained at all and I do not understand anything related to the use of a transfusion. |  |

1. **If your obstetrician felt that a transfusion was necessary for treating anemia after delivery, would you give consent for receiving a blood transfusion?**

Yes / No

If yes, please provide information about why you are happy to receive a blood transfusion:

__________________________________________________________________________________________________________________________________________________________________________________________________________________________________________________________________________________________________________________________________________________________________________________________________________________________

If no, please indicate your reasons for not consenting to receiving a blood transfusion:

__________________________________________________________________________________________________________________________________________________________________________________________________________________________________________________________________________________________________________________________________________________________________________________________________________________________

1. **Were you given information about your last Hemoglobin or Hematocrit blood test taken before your date of delivery?**

YES / NO

If no, go to question 14.

If yes,

Do you know the result of these tests?
YES / NO: If yes, please state this:____________

1. **During your postpartum stay, was a hemoglobin or hematocrit blood test taken after your delivery?**

YES/NO

If yes,

Do you know the result of these tests?
YES/NO: if yes, please state this: _____________
